# Differentially Expressed Circular RNA Profile Signatures Identified in Prolificacy Trait of Yunshang Black Goat Ovary at Estrus Cycle

**DOI:** 10.3389/fphys.2022.820459

**Published:** 2022-04-04

**Authors:** Yufang Liu, Zuyang Zhou, Xiaoyun He, Yanting Jiang, Yina Ouyang, Qionghua Hong, Mingxing Chu

**Affiliations:** ^1^ Key Laboratory of Animal Genetics, Breeding and Reproduction of Ministry of Agriculture and Rural Affairs, Institute of Animal Science, Chinese Academy of Agricultural Sciences, Beijing, China; ^2^ College of Life Sciences and Food Engineering, Hebei University of Engineering, Handan, China; ^3^ Yunnan Animal Science and Veterinary Institute, Kunming, China

**Keywords:** Yunshang black goat, prolificacy trait, estrus cycle, DE circRNA, integrate analysis

## Abstract

CircRNAs acting as miRNA sponges play important roles in the growth process of animal individuals. The prolificacy trait of goats is involved in many pathways, however, the variation of circRNA expression profiles in the different phases of the estrus cycle at high and low fecundity groups is still unknown. Here, we analyzed the circRNA profiles of ovarian tissues among high and low fecundity groups in the follicular phase (HF vs LF), high and low fecundity groups in the luteal phase (HL vs LL), and high and low fecundity in the whole estrus cycle (HF vs HL and LF vs LL) using RNA-seq. A total of 283 (114 upregulated and 169 downregulated), 559 (299 upregulated and 260 downregulated), 449 (254 upregulated and 195 downregulated), and 314 (210 upregulated and 104 downregulated) differentially expressed (DE) circRNAs were screened in HF vs LF, HF vs HL, HL vs LL, and LF vs LL groups, respectively. Enrichment analysis suggested that the targeting of DE circRNAs was mainly enriched in oocyte meiosis, the GnRH signaling pathway, and estrogen signaling pathway. After integrating our previous study of miRNA-seq, there were 56 miRNAs that could target to 192 DE circRNAs, including the miR-133 family (including miR-133a-3p and miR-133b), miR-129-3p, and miR-21, which also had important influence on the prolificacy trait of goats. Then, 18 circRNAs with coding potential were obtained by four software predictions, and 9 circRNAs were validated by RT-qPCR. Together, circRNAs play a key role in the prolificacy trait and the transformation of the follicular phase to the luteal phase in the estrus cycle of goats.

## Introduction

The kidding number of goats, an essential economic indicator of the goat industry, is mainly dependent on ovulation rate and ovarian growth (Notter, 2012). Many factors influence the kidding number and ovulation in goats. The reproductive performance of goats is different to that of sheep, and the genes conferring the major effects on prolificacy of goats are still unknown. Prolific and non-prolific breeds are characterized by differences in the estrus cycle. Determining the molecular mechanisms of follicular development has therefore been a focus of research in the genetic breeding of goats. In recent years, many studies of the mechanisms of prolificacy in goats have focused on candidate genes and their regulators, such as miRNAs, transcription factors, and varieties of regulated pathways. All these factors form a network of many interactions *in vivo* that regulate the goat reproduction trait (Lu et al., 2020; Zhao et al., 2020). The genes GDF9, Smad2, BMP15, and BMP4, and the transcription factor Sox9 are involved in kidding number and increased ovulation rate, and are proven to be crucial for the reproductive performance of goats (Hanrahan et al., 2004; Lai et al., 2016; Chinmoy et al., 2017; He et al., 2020; Wang et al., 2021). In addition, many factors affected the reproductive ability of goats including follicle recruitment, development, selection, and ovulation (Luo et al., 2019). A few signaling pathways, such as the Wnt/β-catenin, nodal mammalian target of rapamycin (mTOR), and bone morphogenetic protein (BMP)/Smad pathways, are involved in follicular development (Li et al., 2016).

CircRNAs are one type of long noncoding RNAs produced by pre-mRNA back-splicing that have been progressively identified and validated to form a covalently closed loop structure of single-strand, non-polyadenylated circular molecules (Memczak et al., 2013; Ashwal-Fluss et al., 2014), which show a previously neglected regulatory method in the form of the circRNA-miRNA-mRNA axes (Hansen et al., 2013). Usually, circular RNAs act as a sponge for related miRNAs, and compared with their corresponding colinear mRNA isoforms, circRNAs are more stable (Chuang et al., 2018). Recently, many studies on circRNA function have shown that regulation of circRNA expression can affect a variety of molecular and physiological phenotypes, mainly related to the nervous system, innate immunity, and diseases (Xiao et al., 2020). CircRNAs also act as a template for translation (Kristensen et al., 2019). Many circRNAs were shown to play important roles in the process of reproduction by regulating the expression of genes at the transcriptional and post-transcriptional levels. In the study of reproduction trait, circRalGPS2s were lowly expressed in follicle growth and predominant in the ovarian stroma, suggesting that circularization of circRNAs may not have a relationship with the development of granulosa cells (Shen et al., 2019). A few studies showed that the expression of circRNA_103827 and circRNA_104816 was negatively correlated with embryonic development (Cheng et al., 2017). Although circRNAs have various functions and act through mechanisms, the regulatory effect of circRNAs on the goat reproduction trait is uncharacterized. Hence, it is crucial to reveal the molecular mechanism of circRNAs regulating the prolificacy trait and the transition from the follicular phase to the luteal phase in goats.

The Yunshang black goat is an excellent breed of meat-producing goat, which has many favorable characteristics including tender meat and unique flavor, and live in Yunnan Province, China. Yunshang black goat is suitable for large-scale breeding in all of regions of China. Many studies have examined the molecular mechanisms of resource conservation, strain selection, and trait formation of this native variety. One excellent feature of Yunshang black goat is prolificacy, making it suitable for the study of prolific goats. The molecular mechanisms underlying prolificacy trait regulation in the Yunshang black goat have not yet been confirmed. In order to reveal the key molecular mechanism networks of the prolificacy trait and follicular development in this variety, in this study, we screened for differentially expressed (DE) circRNAs among high and low fecundity goats in two phases of the estrus cycle (four groups: high fecundity in the follicular phase, high fecundity in the luteal phase, low fecundity in the follicular phase, and low fecundity in the luteal phase, respectively) by RNA-seq. The crucial correlated miRNA-circRNA and circRNA-mRNA regulator networks and pathways in the prolificacy trait and the transition from the follicular phase to the luteal phase were identified. Furthermore, the function and mechanism of DE circRNAs were predicted by functional enrichment and coding potential analysis. These analyses will provide a basis for the molecular mechanism of follicular development and the transition from the follicular phase to the luteal phase in goats. Our purpose was to clarify the molecular mechanisms of follicular phase regulation of goat ovaries and to provide a research basis for further study on the interactions between circRNAs, miRNAs, host genes, and signaling pathways for prolific traits and the transition from the follicular phase to the luteal phase in goats. This study also provides basic data for Yunshang black goat prolificacy trait-related candidate gene screening and the conservation of native breed resources.

## Materials and Methods

### Sample Collection and Preparation

Twenty she-goats (2–3 years old), no significant differences in age and weight, were selected and grouped into four groups, including high fecundity goats in the follicular phase (HF) and in the luteal phase (HL) (*n* = 5, average kidding number ≥3) and low fecundity goats (*n* = 5, average kidding number ≤2, LH and LL groups) based on their kidding number records. All individuals were fed in the same environment and had free access to water in a Yunnan Province goat farm. The ovaries at the follicular phase of the estrous cycle were collected from animals with high (≥3) and low (≤ 2) numbers of kids, frozen immediately in liquid nitrogen, and stored at −80°C for conservation.

### Preparation of RNA Library for Sequencing

According to the manufacturer’s protocol, total RNA was isolated from ground ovarian tissue powder using TRIzol reagent (Invitrogen, Carlsbad, CA, United States ). The degree of degradation and contamination of RNA samples was monitored by 1% agarose gels, and the NanoPhotometer^®^ spectrophotometer (IMPLEN, CA, United States) was used to check the purity of the RNA samples. The Qubit^®^ 2.0 Fluorometer (Life Technologies, CA, United States ) with the Qubit^®^ RNA Assay Kit was used to measure the concentration of RNA. The integrity of RNA was assessed by the RNA Nano 6000 Assay Kit of the Bioanalyzer 2,100 system (Agilent Technologies, CA, United States ).

The 3 μg of RNA per sample was used as the starting material for the RNA samples. Initially, the Epicentre Ribo-zero™ rRNA Removal Kit (Epicentre, United States ) was used to remove rRNA, and ethanol precipitation was used to purify the rRNA free samples. Secondly, the NEBNext^®^ Ultra™ Directional RNA Library Prep Kit for Illumina^®^ (NEB, United States ) was used to deplete RNA from rRNA and generate the sequencing libraries in accordance with the manufacturer. In brief, the divalent cations in NEB Next First Strand Synthesis Reaction Buffer (5X) under elevated temperature were used to perform fragmentation. The random hexamer primer and M-MuLV Reverse transcriptase (RNaseH-) were used to synthesize the first-strand cDNA. Subsequently, DNA polymerase I and rnase H were used to synthesize second-strand cDNA. The dUTP replaced the dNTPs with dTTP in the reaction buffer. The active exonuclease/polymerase converted the remaining overhangs into blunt ends. The 3′-ends of DNA fragment adenylation and the NEB Next Adaptor with hairpin loop structure prepared for hybridization were ligated. The AMPure XP system (Beckman Coulter, Beverly, United States ) was used to purify the library fragments and to select the cDNA fragments with different lengths. PCR was then performed by adding 3 μl of USER Enzyme (NEB, United States ) to size-selected, adaptor-ligated cDNA for 15 min at 37°C, followed by 5 min at 95°C. PCR was subsequently performed with Phusion High-Fidelity DNA polymerase, Index (X), and Primer universal PCR primers. Finally, the Agilent Bioanalyzer 2,100 system was used to assesses library quality, and the AMPure XP system was used to purify the products.

### Identification and Analysis of Differentially Expressed circRNAs

CircRNAs were identified by find_circ and CIRI2 (Memczak et al., 2013; Gao et al., 2017). The following are the basic principles of find_circ: according to Bowtie2 alignment results, find_circ extracted the 20 nt anchor sequences from the ends of the reads that were not aligned to the reference sequence, and aligned each pair of anchor sequences with the reference sequence again. If the 5′-end of the anchor sequence aligned with the reference sequence (start and end sites A3 and A4, respectively), the 3′-end of the anchor sequence was aligned upstream of the site (start and end sites A1 and A2, respectively), and there was a splice over the site (GT-AG) between the A2 and A3 of the reference sequence, then this read could be considered a candidate circRNA. Finally, the candidate circRNAs with read counts greater than or equal to 2 were identified as circRNAs. CIRI2 searched for a PCC (paired end mapping) signal and PEM (pair-end mapping) signal and junction reads of the shear site of paired chiastic clipping according to the comparison of the results of BWA (Li and Durbin, 2009). Then, based on the results of dynamic programming alignment, circRNA read support numbers and genome annotation information was used to filter the candidate circRNAs.

Quantification was performed based on reads that mapped across the circularized junctions and were normalized using SRPBM (number of circular reads/number of mapped reads (units in billion)/read length) (Zheng et al., 2016). Differential circRNA expression was analyzed using DESeq2. CircRNAs with *padj* values of < 0.05 were considered to be significantly differentially expressed.

### The GO and KEGG Enrichment of Host Genes With DE circRNAs

Functional annotation of host genes with DE circRNAs was based on the GO and KEGG annotations of the source genes. GO annotation was performed based on the corresponding genes in NCBI and their GO a nnotations. This information was held in the following database https://ftp.ncbi.nlm.nih.gov/gene/DATA/gene2go.gz. For KEGG annotation, the KOBAS software was used to test the statistical enrichment of host genes with DE circRNAs (Xie et al., 2011).

### Prediction Analysis of the Targeting miRNA of DE circRNAs

CircRNAs, a type of targeting molecule of miRNAs, are regulated by miRNA. miRanda was used to predict the miRNA interaction sites on circRNA for animals (Fahlgren and Carrington, 2010). The function and mechanism of action of circRNAs as miRNA sponges were investigated through the analysis of circRNA-miRNA interactions.

### Coding Potential Prediction of circRNAs

CircRNAs have the potential to encode protein, and in this study IRESfinder software was used to calculate the IRES score for each circRNA. The score of >0.5 indicated the presence of IRES elements, with a higher score indicating higher accuracy. The coding potential of the circRNA sequences were further identified by the CPC2, CNCI, CPAT, and PLEK software programs.

### Verification of Sequencing Results by RT-qPCR

Several DE circRNAs were randomly selected to verify the reliability of the sequencing data, and confirmed by RT-qPCR with RPL19 used as the reference gene. Accounting for circRNA characteristics, RT-qPCR primers were designed, and relevant primer information is shown in Table 1. Three duplicates per sample were analyzed, and a RocheLight Cycler^®^480 Ⅱ system (Roche Applied Science, Mannheim, Germany) was used to establish a standard curve. The following steps were applied for RT-qPCR: initial denaturation at 95 °C for 5 min, followed by 40 cycles of denaturation at 95 °C for 5 s, and annealing at 60 °C for 30 s. The data were analyzed using the 2^*-∆∆Ct*^ method (Liu et al., 2021). The relative expression was calculated by a dependent sample *t*-test and SPSS 19.0 was used to analyze the significance of any differences.

**TABLE 1:** The primer information of DE circRNAs.

ciRNA ID	Primer Sequence (5′-3′)	Product Size (bp)	Tm (°C)
circUCHL3	F: GCA​TCC​TAA​CTG​GCA​GTT​TGT​T	126	60
	R: ACA​ATG​CTG​AGC​TCA​TCC​TTT​T		
circTIAM1	F: TGG​AAG​ATG​GAG​TGA​GAT​TGG​T	208	60
	R: CAG​ATA​ACC​TTG​CGC​AGC​TT		
circRAD51C	F: CTG​CAC​TGG​GGG​AAA​GTT​G	154	60
	R: CTG​CAA​GGC​AAC​AGA​GAT​GG		
cicrPUM2	F: TTT​TGT​TCT​CCG​TGC​TGG​G	201	60
	R: CTC​CCA​TTC​CCC​GAG​ATT​C		
circOSBPL1A	F: ACC​ACG​CAC​TCC​TTT​GAC​G	181	60
	R: GTC​GCC​AGC​GCT​TCT​GAT​A		
circMTCL1	F: AAC​CGT​GGA​GGA​AAA​GAG​AGC	158	60
	R: TCA​TCT​CTT​CCA​TTT​CCG​CTC		
circITK	F: CTC​CCG​AAT​CAA​ATG​TGT​CG	117	60
	R: CGG​TCT​GGA​GCA​AAC​ACA​TAC		
circFARS2	F: TTT​GGA​GAC​GGA​CTG​GAC​ATC	140	60
	R: GAG​TTT​ACC​AGC​TGC​TGC​TCC		
circAKT3	F: GGA​GAG​GAA​GAG​ATG​GAT​GCT​T	128	60
	R: GTG​TTT​GGC​TTT​GGT​CGT​TCT		
RPL-19	F: ATCGCCAATGCCAACTC	154	60
	R: CCT​TTC​GCT​TAC​CTA​TAC​C		

### PPI Analysis of Host Genes

As the goat database is not complete, we used the database of sheep, which has a high homology with goats, as a reference. The protein-protein interaction (PPI) analysis of host genes of DE circRNAs was conducted according to the STRING database (Organism: *Ovis aries*). We constructed the PPI network by extracting a list of host genes from the sheep database. In this study, the online tool STRING was used analyze the PPI of host genes, and PPIs with a score of >700 were selected as significant interactions.

## Results

### Analysis of Transcriptome Sequencing of Ovaries in Yunshang Black Goats

**FIGURE 1:**
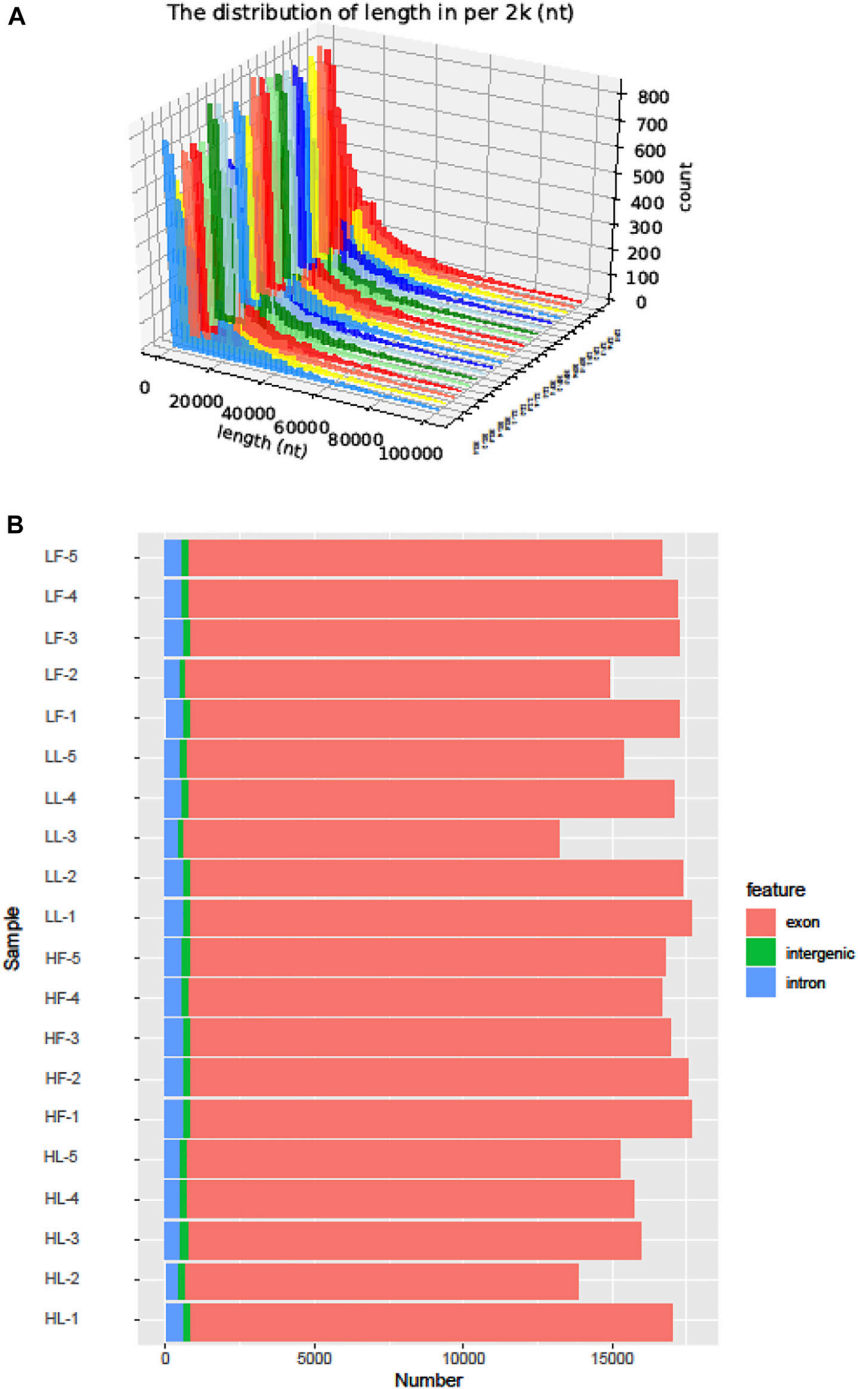
The identification of circRNAs in goat ovarian tissues. **(A)**: Length distribution of circRNAs. **(B)**: Function region statistics of circRNAs.

**FIGURE 2:**
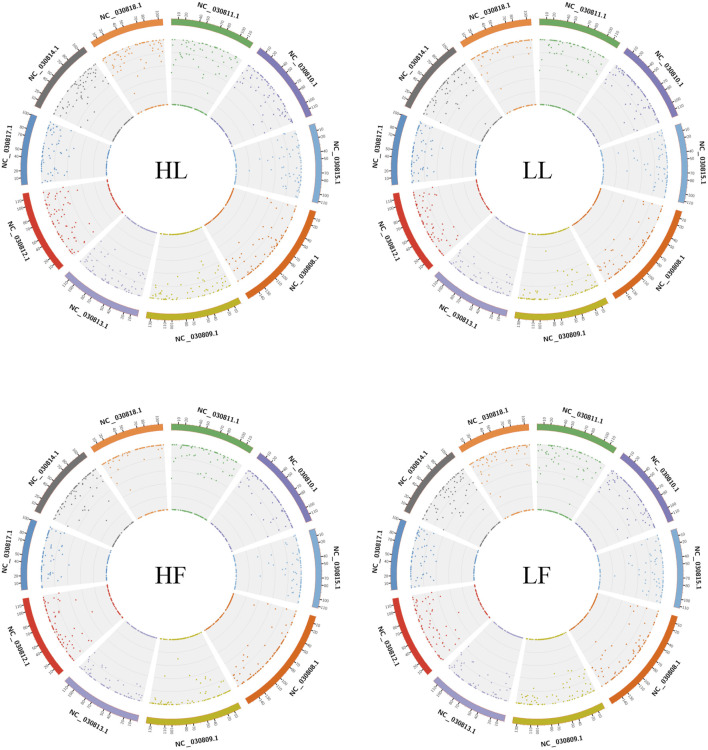
Density statistics of circRNAs in chromosomes in the four groups. HL: high fecundity goats in the luteal phase; LL: low fecundity goats in the luteal phase; HF: high fecundity goats in the follicular phase; LF: low fecundity goats in the follicular phase.

Twenty cDNA libraries of ovaries from high and low fecundity goats in the follicular and luteal phases were constructed, and an Illumina Hiseq 2,500 sequencer was used to perform the sequencing. The samples were divided into four groups, and five replicates of each sample were considered: the high fecundity group in the follicular phase (HF-1, HF-2, HF-3, HF-4, HF-5), the high fecundity group in the luteal phase (HL-1, HL-2, HL-3, HL-4, HL-5), the low fecundity group in the follicular phase (LF-1, LF-2, LF-3, LF-4, LF-5), and the low fecundity group in the luteal phase (LL-1, LL-2, LL-3, LL-4, LL-5). In Table 2, the number of clean reads and GC contents for the four groups are shown. The average rate of clean reads that uniquely mapped to the *Capra hircus* genome was 90.80% (from 87.16 to 94.36%). After mapping the reference sequence, we identified 6,034 circRNAs. The spliced length distribution of circRNAs was primarily in the range from 200 bp to 10,000 bp (Figure 1A). CircRNAs are mainly derived from exon splicing, with smaller proportions arising from intron and intergenic splicing (Figure 1B). The circRNA density statistics for each chromosome showed that circRNAs were distributed on chromosome 1 to 11, with the highest proportion of circRNAs located on chromosome 1, 2, and 3 (approximately 32%) (Figure 2 and Supplementary Figure S1). In addition, circRNAs were named after the host genes (Supplementary Table S1).

**TABLE 2:** Summary of data quality and genome alignment.

Sample Name	Raw Reads	Clean Reads	GC Content (%)	Uniquely Mapped	Aligned Rate (%)
HF-1	109,570,172	105,954,528	51.65	96,068,550	87.68
HF-2	148,187,868	142,902,849	48.89	135,791,218	91.63
HF-3	1,64,219,488	158,462,267	48.84	150,443,597	91.61
HF-4	99,578,072	96,294,943	52.72	86,789,451	87.16
HF-5	100,348,242	96,908,787	49.66	9,1,351,998	91.03
HL-1	108,208,302	105,414,299	48.96	99,141,316	91.62
HL-2	120,615,862	117,258,743	51.65	106,634,997	88.41
HL-3	106,147,406	103,016,047	51.32	95,130,195	89.62
HL-4	100,702,026	98,039,488	50.62	91,299,898	90.66
HL-5	105,616,808	102,473,442	44.00	98,955,302	93.69
LF-1	102,208,602	99,264,769	46.21	95,507,767	93.44
LF-2	128,736,444	119,602,467	47.61	114,010,299	88.56
LF-3	102,912,346	100,075,443	50.49	93,100,212	90.47
LF-4	124,494,838	120,693,801	50.90	111,591,728	89.64
LF-5	103,708,734	100,662,136	50.16	93,921,221	90.56
LL-1	106,458,938	103,537,898	45.44	100,451,466	94.36
LL-2	107,178,306	104,468,502	49.39	98,751,596	92.14
LL-3	139,843,390	135,000,615	49.08	127,584,232	91.23
LL-4	145,089,226	139,603,939	48.99	131,614,325	90.71
LL-5	114,365,628	110,768,994	49.56	104,849,118	91.68

### Analysis of the Expression of circRNAs

**FIGURE 3:**
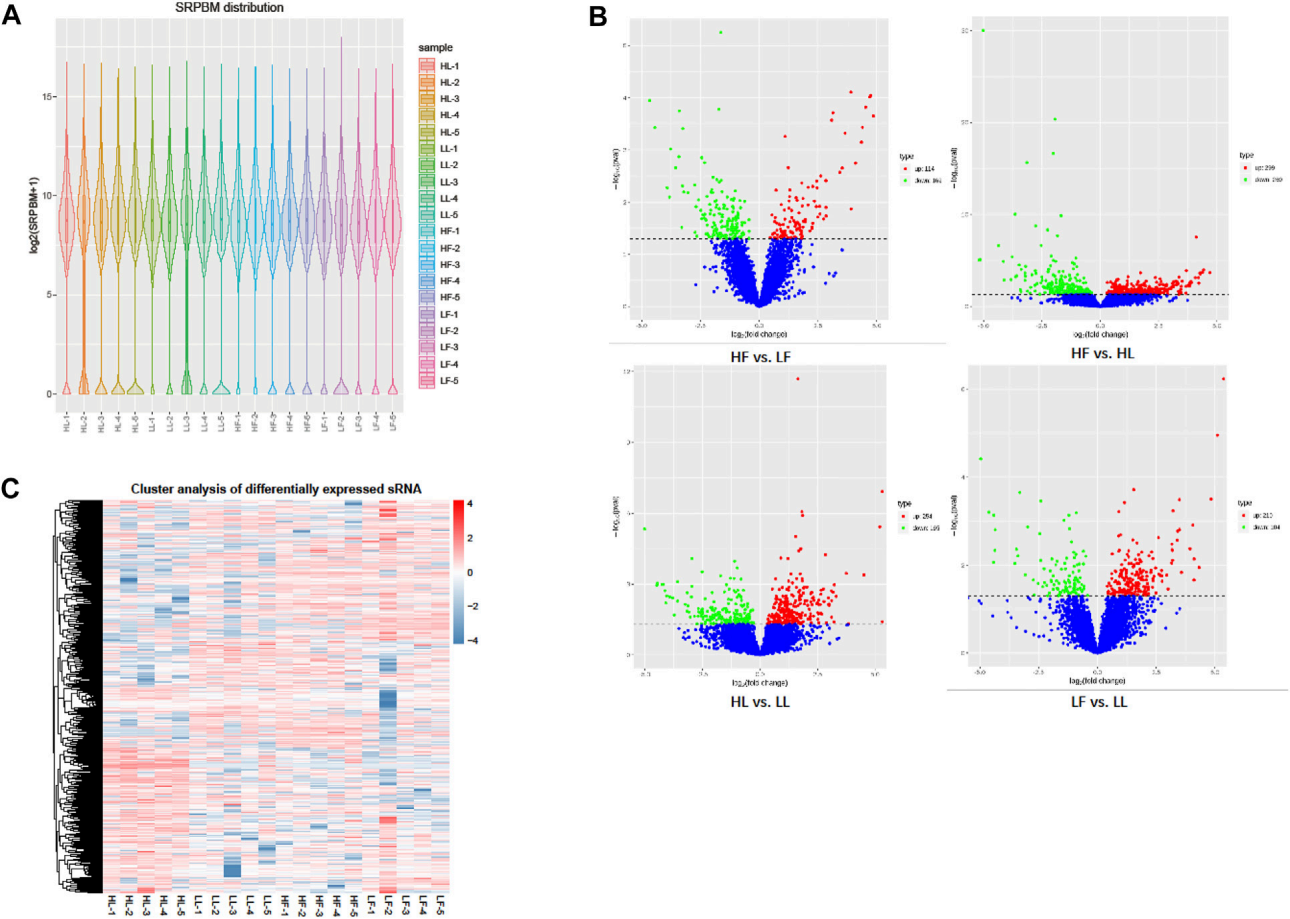
DE circRNA screening and clustering analysis. **(A)**: The distribution of circRNA expression levels in each sample. The expression of circRNAs was normalized by SRPBM. **(B)**: The volcano plot of DE circRNAs in the four comparisons. The *X*-axis represents the change in circRNA expression in different comparisons. The *Y*-axis represents the statistical significance of circRNAs. The blue dots represent the circRNAs that were not significantly different, the red dots represent circRNAs that were significantly upregulated, and the green dots represent circRNAs that were significantly downregulated. **(C)**: The clustering diagram of DE circRNAs. The expression was gradually upregulated from blue to red.

**FIGURE 4:**
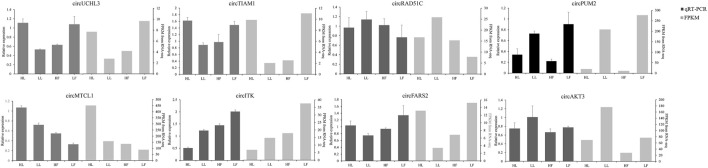
Comparison of the results of DE circRNAs in RNA-seq and RT-qPCR analysis.

The expression of circRNAs per individual was calculated, and their levels were normalized to SRPBM (Figure 3A). Based on the normalized expression with |log2 (fold change) | > 1 and *p*-value < 0.05, 283 DE circRNAs (114 upregulated and 169 downregulated), 559 DE circRNAs (299 upregulated and 260 downregulated), 449 DE circRNAs (254 upregulated and 195 downregulated), and 314 DE circRNAs (210 upregulated and 104 downregulated) were identified in the HF vs LF, HF vs HL, HL vs LL, and LF vs LL comparisons, respectively (Figure 3B, Supplementary Table S1). The result showed that the host genes of circPRLR, circTGFBR2, and circFSHR were PRLR, TGFBR2, and FSHR, respectively. PRLR and FSHR are important genes in ovulation and these circRNAs probably related to follicular growth and development, or even to the goat prolificacy trait. To analyze the clustering model of the four groups with DE circRNAs, K-means and SOM clustering analyses were used to clustered the 1605 DE circRNAs (Figure 3C). For further validating the reliability of circRNA results, nine DE circRNAs were randomly selected for RT-qPCR across the four groups. The RT-qPCR results accorded with the RNA-seq data (Figure 4).

### Enrichment Analysis of the Host Genes for DE circRNAs

**FIGURE 5:**
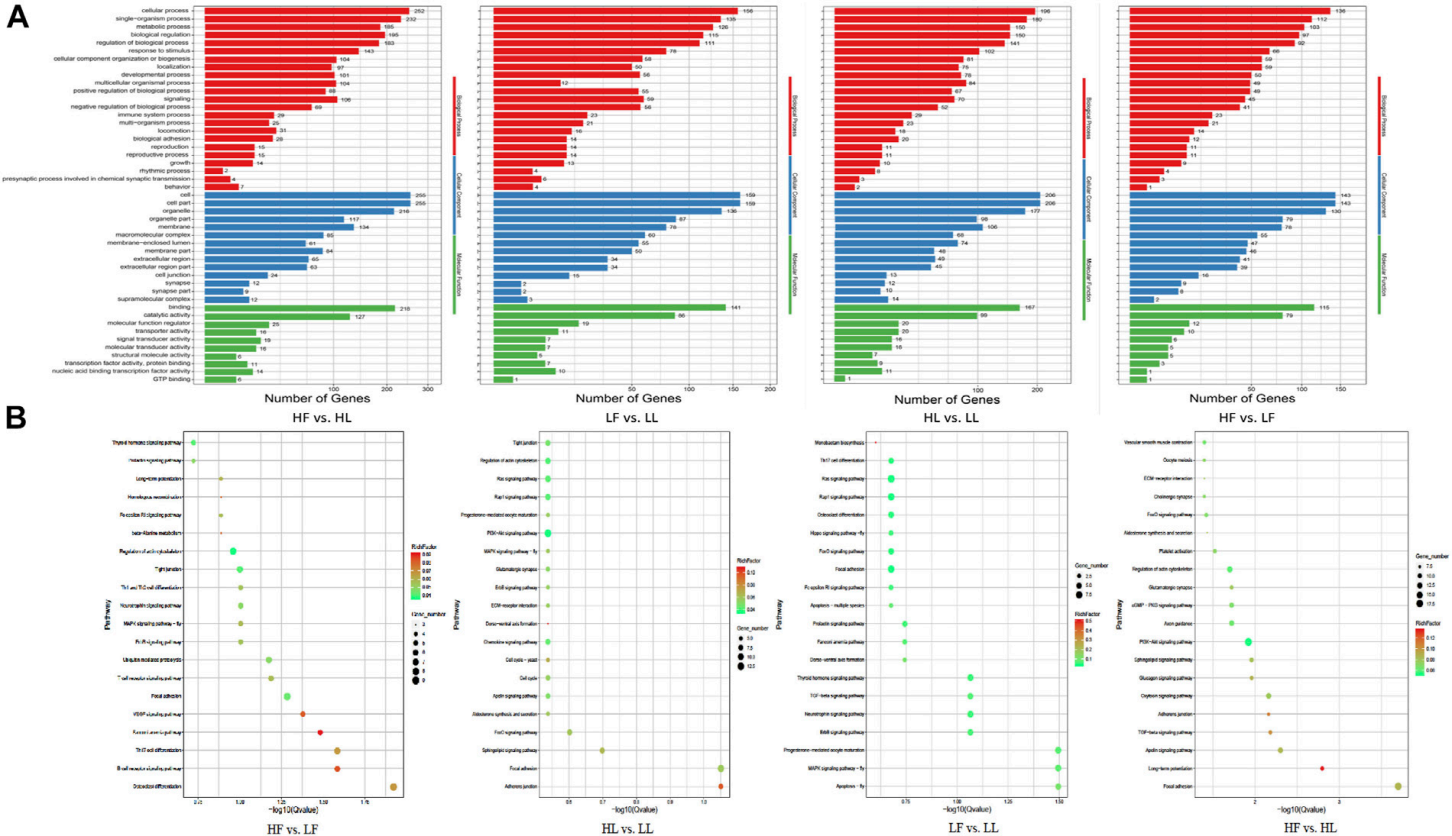
GO and KEGG enrichment analysis of the host genes with DE circRNAs. **(A)**: The results of GO enrichment. **(B)**: The KEGG pathway enrichment.

GO and KEGG enrichment analyses of host genes for DE circRNAs were performed using the appropriate online databases. GO terms analysis showed that there were 558 host genes with DE circRNAs in the HF vs HL comparison, which were annotated into 4,607 functional subclasses with 814 significant GO items. In this comparison, the most significantly enriched GO terms were in cell and cellular process subclasses. In the LF vs LL comparison, a total of 3,684 functional subclasses and 492 terms were significant; most enrichment occurred in cellular component (CC) subclasses. Moreover, in the HF vs LF comparison, terms were related to cell part and cell terms. There were 3,643 enriched terms in this comparison, with 811 terms significantly enriched. In the HL vs LL comparison, we found 4,377 terms were enriched, 571 of which were significantly enriched. Of which, a lot of host genes for DE circRNAs were enriched in the cell (GO:0005623), cell part (GO:0044464), and cellular process (GO:0009987) subclasses, along with other subclasses (Figure 5A). Our results suggested that some GO terms associated with cellular process showed the highest value in the HF vs HL comparison, followed by that in HL vs LL, LF vs LL, and HF vs LF. However, for almost all reproduction-related GO terms, such as reproductive process (GO:0022414), ovulation cycle (GO:0042698), oogenesis (GO:0048477) and sexual reproduction (GO:0019953), the enrichment in the HL vs HF comparison was higher than in the LL vs LF comparison. We speculated that the host gene enrichment in these GO terms was associated with the transformation of the follicular phase to the luteal phase. In the HF vs LF and HL vs LL comparisons, the GO terms were more focused on reproductive process (GO:0022414), hormone receptor binding (GO:0051427), steroid metabolic process (GO:0008202), and ovulation cycle process (GO:0022602). These results showed that there were differences in the regulation of biological processes related to reproductive processes and hormone secretion in the different comparisons.

Comparison of the host genes and KEGG pathway analysis showed that the host genes were annotated into 154, 175, 157, and 183 pathways for the HF vs LF, HL vs LL, LF vs LL, and HF vs HL comparisons, respectively (Figure 5B). Of these, progesterone-mediated oocyte maturation (ko04914), ovarian steroidogenesis (ko04913), oocyte meiosis (ko04114), the GnRH signaling pathway (ko04912), the estrogen signaling pathway (ko04915), and other pathways were involved in the development of ovarian and oocyte maturation.

### Analysis of Targeting Relationship Between circRNAs and miRNAs

**FIGURE 6:**
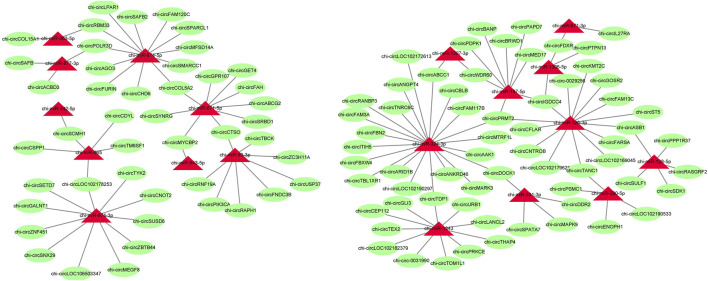
Predicted target relationships of circRNAs and miRNAs. Red triangles represent miRNAs. Green circles represent circRNAs.

To further identify the key circRNAs in the prolificacy trait of goats, the binding sites of miRNA-bound circRNAs were predicted by miRanda software. We found that 56 miRNAs bind to 192 DE circRNAs. Of note, our result showed that the relationship of miRNA-circRNA showed the one-to-many and many-to-one characteristic (Figure 6, Supplementary Table S2). miR-133a-3p, miR-133b, miR-129-3p, and miR-21 were found, which mainly have an impact on ovary development, thus, their target circRNAs were likely to be associated with ovary development.

### Identifying the Coding Potential of circRNAs

Among the four groups, in total, 4083 DE circRNAs were calculated with scores greater than 0.5 by IRESfinder software. Furthermore, four software programs were used to predict the coding potential of the above circRNAs, and 3,257 circRNAs were found. Finally, 18 circRNAs were identified with coding potential by the four software programs mentioned above (Table 3).

**TABLE 3:** The predicted results for the coding potential of circRNAs.

circRNA ID	IRES_Score	CPC2	CNCI	CPAT	PLEK
circKAT2B	0.51	TRUE	TRUE	TRUE	TRUE
circSENP5	0.51	TRUE	TRUE	TRUE	TRUE
circUSP24	0.61	TRUE	TRUE	TRUE	TRUE
circASB1	0.53	TRUE	TRUE	TRUE	TRUE
circWASL	0.51	TRUE	TRUE	TRUE	TRUE
circCPSF6	0.57	TRUE	TRUE	TRUE	TRUE
circSLC12A2	0.72	TRUE	TRUE	TRUE	TRUE
circXPA	0.63	TRUE	TRUE	TRUE	TRUE
circSMC6	0.54	TRUE	TRUE	TRUE	TRUE
circPDS5B	0.58	TRUE	TRUE	TRUE	TRUE
circNBEA	0.57	TRUE	TRUE	TRUE	TRUE
circFAM188A	0.85	TRUE	TRUE	TRUE	TRUE
circISM1	0.51	TRUE	TRUE	TRUE	TRUE
circEIF3E	0.77	TRUE	TRUE	TRUE	TRUE
circZCCHC8	0.68	TRUE	TRUE	TRUE	TRUE
circANKRD27	0.52	TRUE	TRUE	TRUE	TRUE
circVRK1	0.52	TRUE	TRUE	TRUE	TRUE
circEIF4E3	0.59	TRUE	TRUE	TRUE	TRUE

### PPI Network for Goat Prolificacy Trait

**FIGURE 7:**
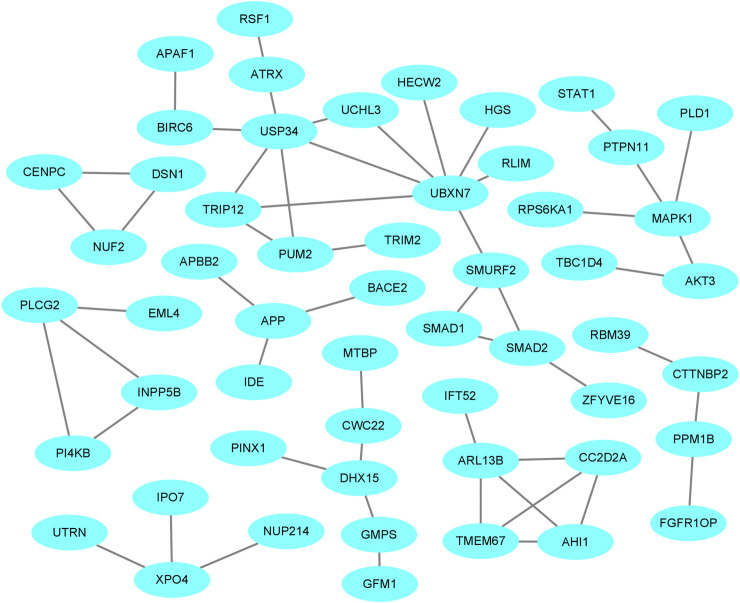
The protein-protein interaction (PPI) network of host proteins with DE circRNAs in the comparisons.

The PPI network of the host genes was constructed by Cytoscape using the analysis result of host genes from the STRING database (Figure 7). For the four comparisons, the PPI network contained 69 protein-protein pairs. MAPK1, which has three nodes including MAPK1-PRS6KA1, MAPK1-PTPN11, and MAPK1-PLD1, is associated with ovarian development and diseases. Other pairs were known to influence the growth and development of the ovary and the prolificacy trait of mammals.

## Discussion

The process of reproduction is very complex. In particular, the study of the goat reproductive molecular mechanism is still in its infancy and there are still many unanswered questions. The reproduction traits in goats are governed by the precise regulation of genes, regulatory elements, and other factors, such as spatiotemporal expression, transcriptional regulation, and feedback mechanisms (Ye et al., 2018; Luo et al., 2019). Investigation of the ovary from goats in different estrus stages should provide information on potential follicle development and allow further improvement to kidding numbers. In a previous molecular biology study, hormone secretion and cell signaling transduction were found to play an important role in the mechanisms of ovarian and follicular development (Messina et al., 2016; Zou et al., 2020). Recently, circRNAs were reported to have a range of extremely important effects on many kinds of biological processes (Memczak et al., 2013). A lot of studies have showed that circRNAs are widespread in plants and mammals, however, little is known about circRNAs in goats, especially those related to the prolificacy trait and ovarian development during the different phases of the estrus cycle (Rybak-Wolf et al., 2015; Ye et al., 2015).

Here, the high-throughput sequencing of circRNAs in the ovaries from goats with different kidding number records was performed in different estrus phases. The circRNAs were distributed widely among chromosomes 1–11 in the four comparisons, and which showed that circRNAs had complexity and functional diversity (Figure 2, Additional file Supplementary Figure S1). The majority of circRNAs in this study were <10,000 nt in length, which was longer than those in previous studies in chicken granulosa cells (200–300 nt) (Shen et al., 2019). In total, 53.8% of the circRNAs were shorter than 10,000 nt and longer than 1,000 nt, the proportion was also higher than that in the studies of goat endometrium circRNAs (23% > 1,000 nt) (Song et al., 2019). Species and tissue differences may be the main reason for the variation in circRNA length. In our study, the main source of circRNAs in the ovary was exon splicing, which was supported by previous research (Lv et al., 2020). Given all of the above features, it was concluded that there were species- and tissue-specific differences in circRNA expression. The changes in circRNA abundance in the four comparisons might be associated with their specific roles during the different estrus phases of the ovary and kidding numbers. We identified 283, 559, 449, and 314 DE circRNAs in HF vs LF, HF vs HL, HL vs LL, and LF vs LL comparisons, respectively (Figure 3B, Supplementary Table S1). Then, these host genes of DE circRNAs were analyzed for GO terms and KEGG pathways. The majority of enriched GO terms were related to cell proliferation and reproductive process. There were 14 host genes of circRNAs related to the reproductive process; notably, mitogen-activated protein kinase 1 (MAPK1), which could mediate luteinizing hormone-induced communication disruption and oocyte maturation in ovarian follicles of rat (Gratao et al., 2008). MAPK1 was shown to play a basic role in regulating spindle assembly, microtubule organization, chromosome distribution, and meiosis resumption, and it could be hypothesized that activation of MAPK1 might improve meiosis resumption (Gordo et al., 2001). MAPK1 mRNA plays a crucial role in oocyte maturation in various species, including cattle and dogs, through its action on granulosa and cumulus cells (Quetglas et al., 2010; Lee et al., 2017). Moreover, some other reproduction-related pathways were also significantly enriched, including oocyte meiosis, the GnRH signaling pathway, and the estrogen signaling pathway.

In the HF vs LF comparison, circIGF2R, circMAPK8, circMYH10, and circPTEN were associated with the prolificacy trait. The IGF2R locus contains a highly abundant set of circIGF2R circRNAs, that showed multiple forms of different exon combinations in mice and plays significant roles in mammalian reproduction (Perera et al., 2018). MAPK8 might regulate the transcription of basal FSHb, and one study has shown that blocking its activation could inhibit the levels of major basal FSHb transcripts by half (Haisenleder et al., 2008). In the HL vs LL comparison, the host genes of circFSHR, circHMGCR, circSMAD3, and circFGFR2 were FSHR, HMGCR, SMAD3, and FGFR2, respectively. FSHR stimulates Gαs protein-dependent activation of cAMP/PKA, leading to phosphorylation of cAMP response element binding protein and steroidogenic protein, which is required for the production of estrogens, which in turn is known to be a growth stimulant (Casarini and Crépieux, 2019). In a study of common genetic variants affecting dizygotic (DZ) twins, a GWAS conducted in 1980 mothers of spontaneous DZ twins and 12,953 controls found that SNPs similar to SMAD family member 3 (SMAD3) were significant associated with DZ twinning (Mbarek et al., 2016). Collectively, these results suggested that those circRNAs were involved in ovarian and oocyte development and thus influence the number of kidding.

Among the DE circRNAs of the comparison between the estrus phases (HF vs HL and LF vs LL), we found that the host genes of circLDLR, circMAPK1, circSMAD1, and circSMAD2 were LDLR, MAPK1, SMAD1, and SMAD2, respectively. SMAD1 and SMAD2 are members of the SMAD family, which is related to signal transduction in the TGF-β superfamily and the inhibition of BMP activity, and they have been thought to control follicular selection by balancing luteinizing hormone receptor (LHR) transcription (Yu et al., 2017; Xu et al., 2018). In addition, LDLR has been shown to take part in the biosynthesis of steroid hormones by affecting the absorption of cholesterol substrates via circulating lipoproteins (Ying et al., 2013). The MAPK1 (p42ERK2) isoform of MAPK is found in mammalian oocytes and the kinase is shown to be activated during meiotic maturation (Fan and Sun, 2004). Together, these circRNAs might be associated with the difference in the estrus phases. In previous studies, similar results were found for non-coding RNAs, including long non-coding RNA and microRNA enrichment analysis, suggesting that these circRNAs might act as sponges of endogenous RNAs (ceRNAs) to regulate gene expression (Kutzler et al., 2001). The results obtained from this study and previous reports suggested that the prolificacy trait and estrus induction are complex processes that includes numerous events, and that the circRNAs distributed in the two estrus phases in high and low fecundity goats are mainly involved in follicle growth, reproduction events, and other biological processes.

Prediction of circRNA-miRNA binding relationships was completed by the miRNA target gene prediction method. In total, binding was predicted for 56 miRNAs, including the miR-133 family (such as miR-133a-3p and miR-133b), miR-129-3p, and miR-21. Several of these miRNAs have been shown to be closely related to ovarian development and the prolificacy trait. Differential expression of miR-133 occurs during meiotic maturation of oocytes (Song et al., 2014). MiR-129-3p serves as a tumor suppressor by targeting BZW1 in ovarian cancer cells and the restoration of miR-129-3p might be a novel therapeutic strategy for ovarian cancer (Liu et al., 2018). MiR-21 promotes follicle development in the ovary during ovulation in mice (Carletti et al., 2010). In the chicken reproductive process, circRalGPS2 can compete with miRNA to modulate mRNA expression that regulates downstream target genes (Chiu et al., 2017). In our study, miR-129-3p was predicted to target chi-circC2H2orf76, chi-circDUS2, chi-circGPR137B, chi-circMYSM1, and chi-circSVEP1, miR-21-3p was predicted to targeting chi-circUTP20, and miR-133a-3p and miR-133b were predicted to targets to chi-circAKAP10. Overall, these differentially expressed circRNAs may regulate the function of the ovary, for estrus and different kidding numbers, by regulating miRNA, although specific mechanisms require further study.

As circRNAs have a loop-like structure consisting of exons, some circRNAs consist of an open reading frame and IRES. Therefore, researchers hypothesized that numerous circRNAs could be translated into protein in certain conditions (Nagarjuna et al., 2017). IRESfinder, CPC2, CNCI, CPAT, and PLEK software identified 18 circRNAs with coding potential. It should be noted that the biological function of these circRNAs, from the perspective of protein translation, should be explored further. In addition, we demonstrated the interactions between host genes in the ovary of Yunshang black goats. A total of 69 PPI pairs for the comparisons and several host genes were associated with the prolificacy trait. The relationships between these interactions require further study.

## Conclusion

In summary, 283 (114 upregulated and 169 downregulated), 559 (299 upregulated and 260 downregulated), 449 (254 upregulated and 195 downregulated), and 314 (210 upregulated and 104 downregulated) DE circRNAs were identified in HF vs LF, HF vs HL, HL vs LL, and LF vs LL comparisons, respectively. Enrichment analysis showed that a number of host genes with DE circRNAs were enriched in oocyte maturation, ovarian steroidogenesis, oocyte meiosis, and the GnRH signaling pathway, which have important impacts on follicular growth and development. In addition, 192 DE circRNAs were found to target 56 miRNAs, 18 circRNAs had coding potential, and 69 PPI pairs were found. These results provide fundamental data on the molecular mechanisms underlying the prolific trait in goats.

## Data Availability

The datasets presented in this study can be found in online repositories. The name of the repository and accession number can be found below: SRA, NCBI; PRJNA726268.
