# Supplementary figures and images for: Differentially Expressed Circular RNA Profile Signatures Identified in Prolificacy Trait of Yunshang Black Goat Ovary at Estrus Cycle
Source: Front Physiol. 2022 Apr 4;13:820459. doi: 10.3389/fphys.2022.820459 (PMC9049588; doi:10.3389/fphys.2022.820459)

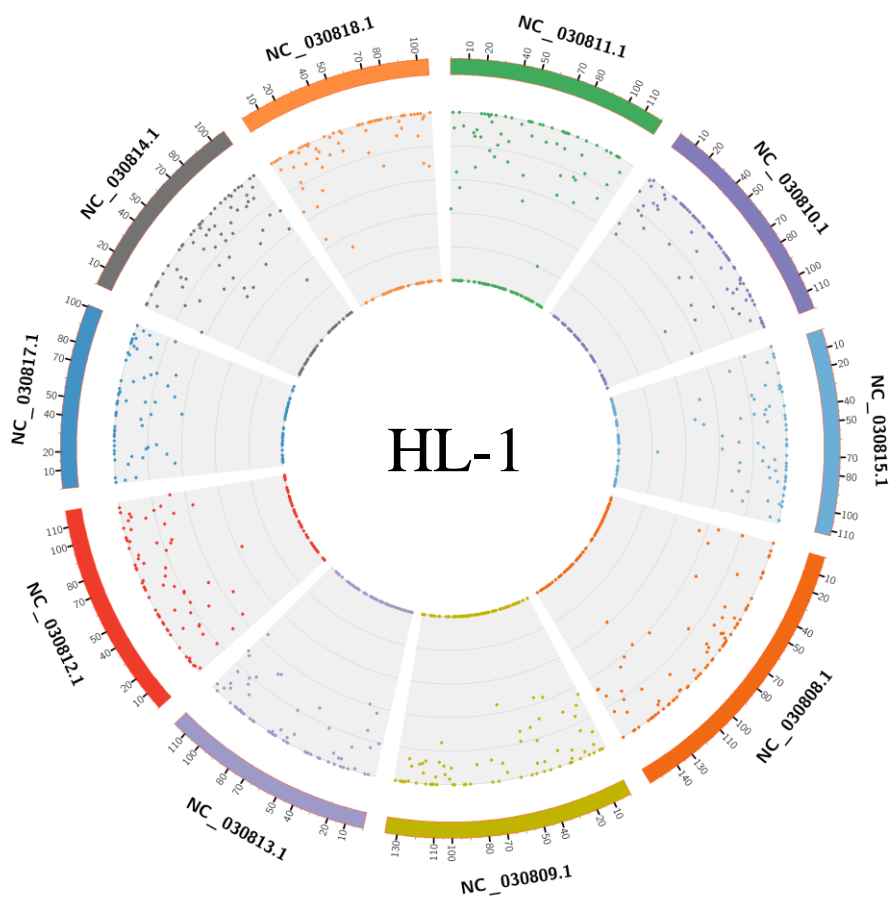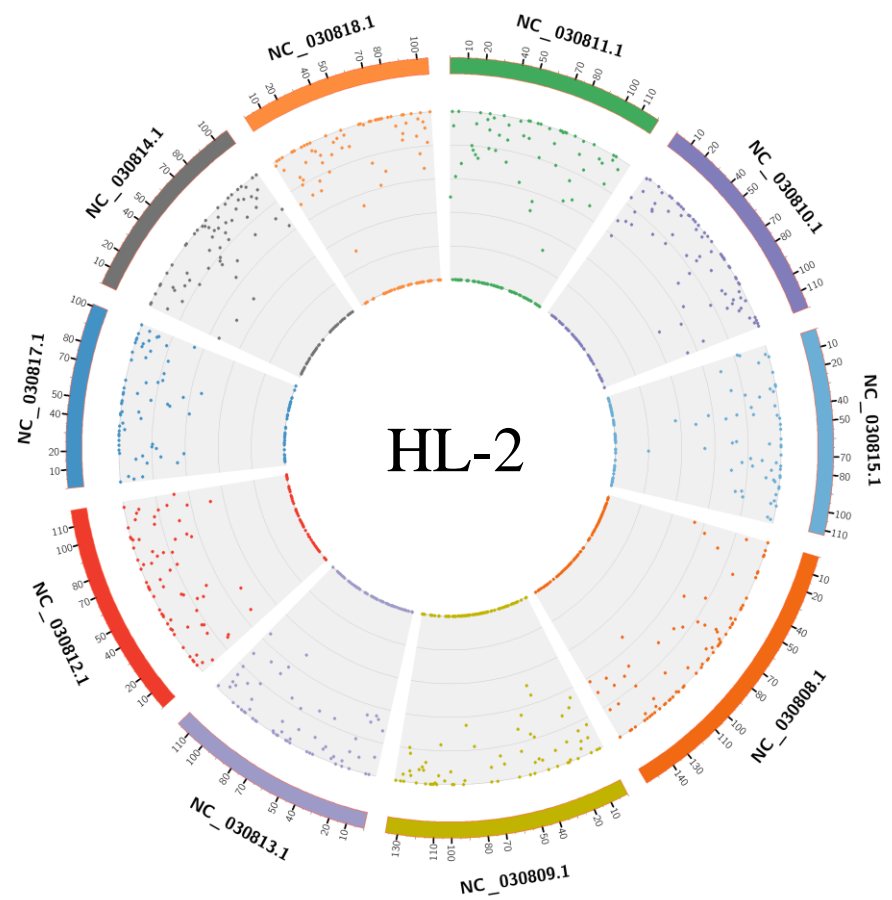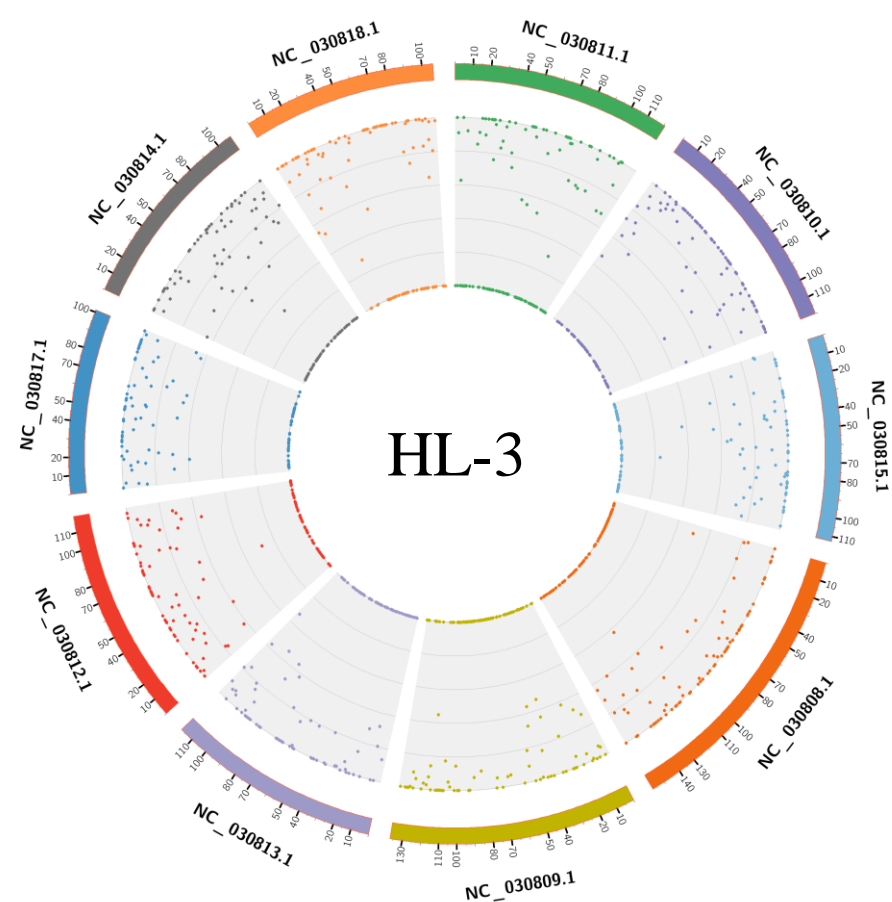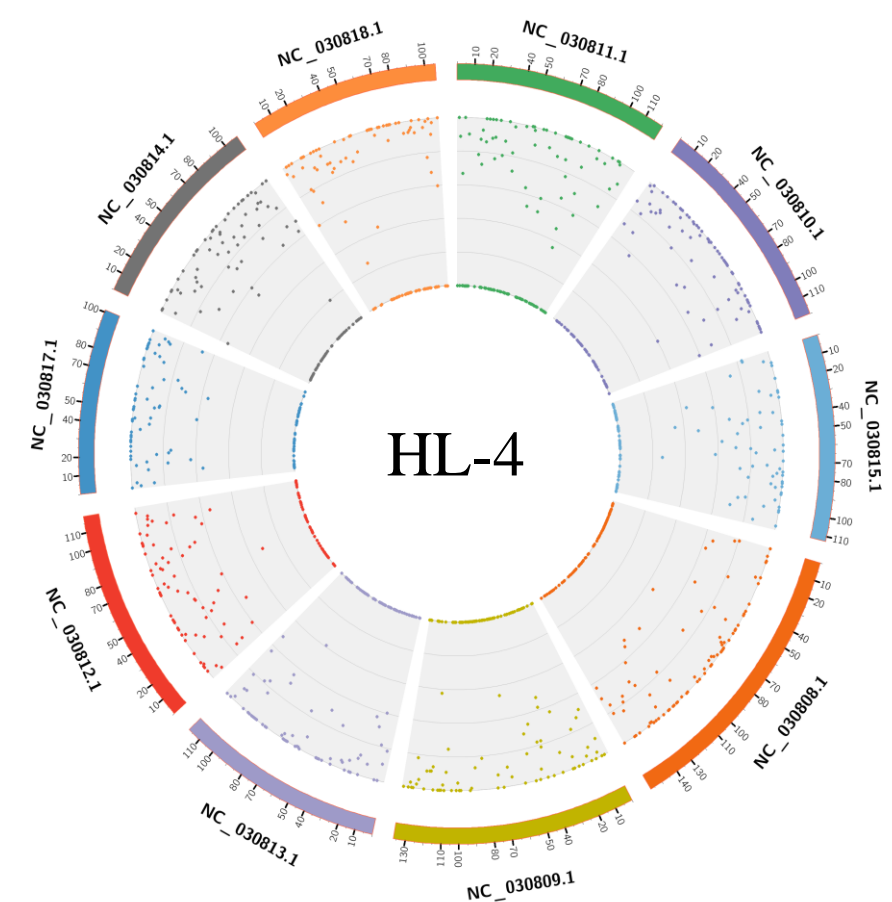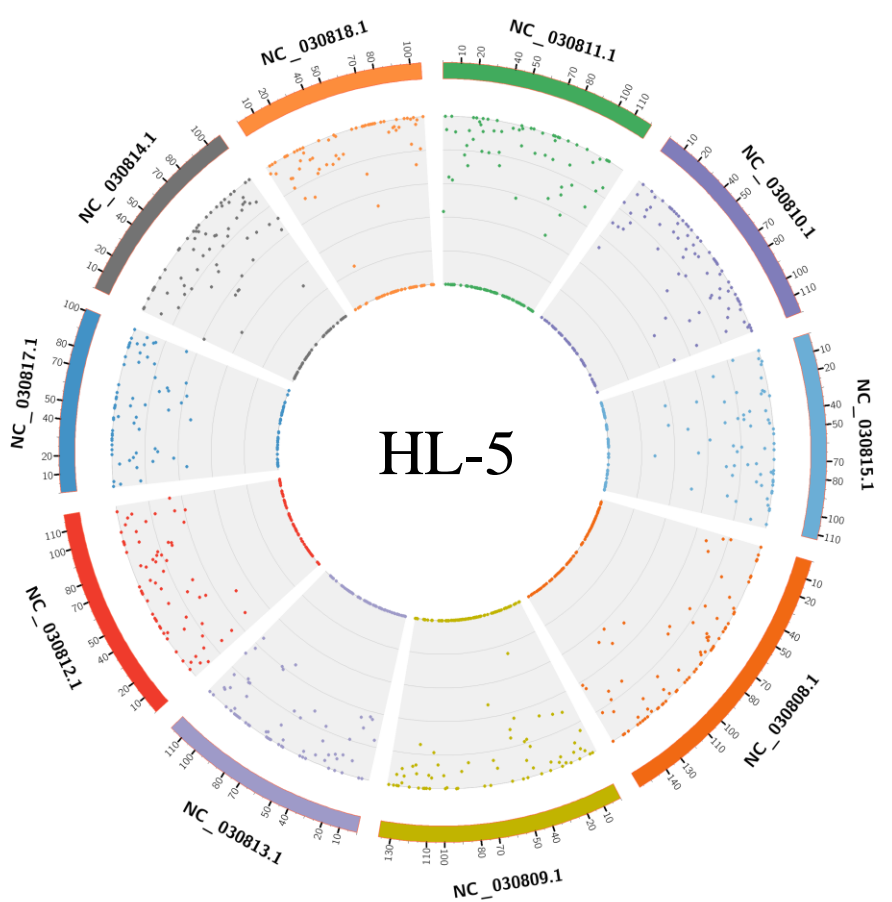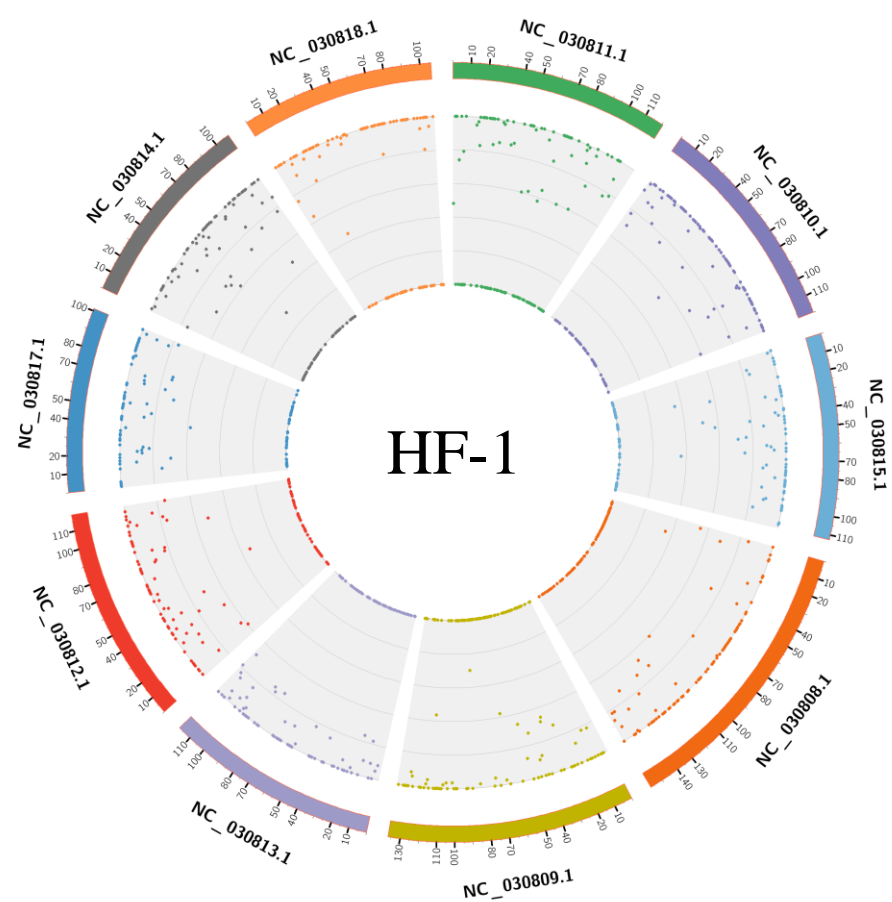

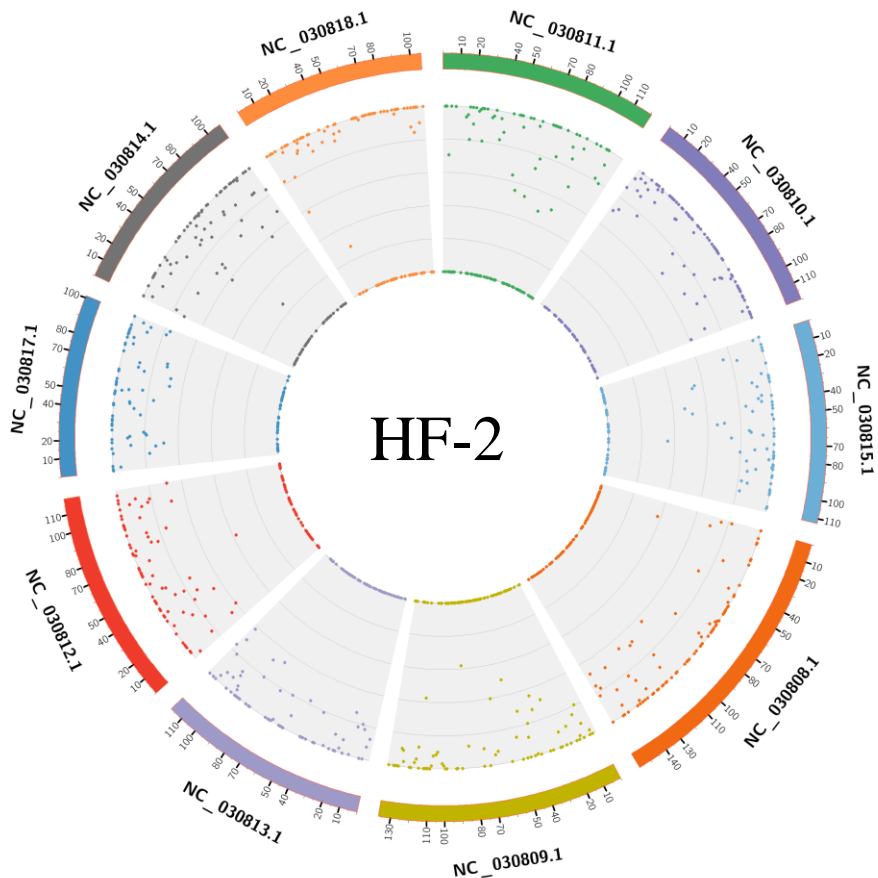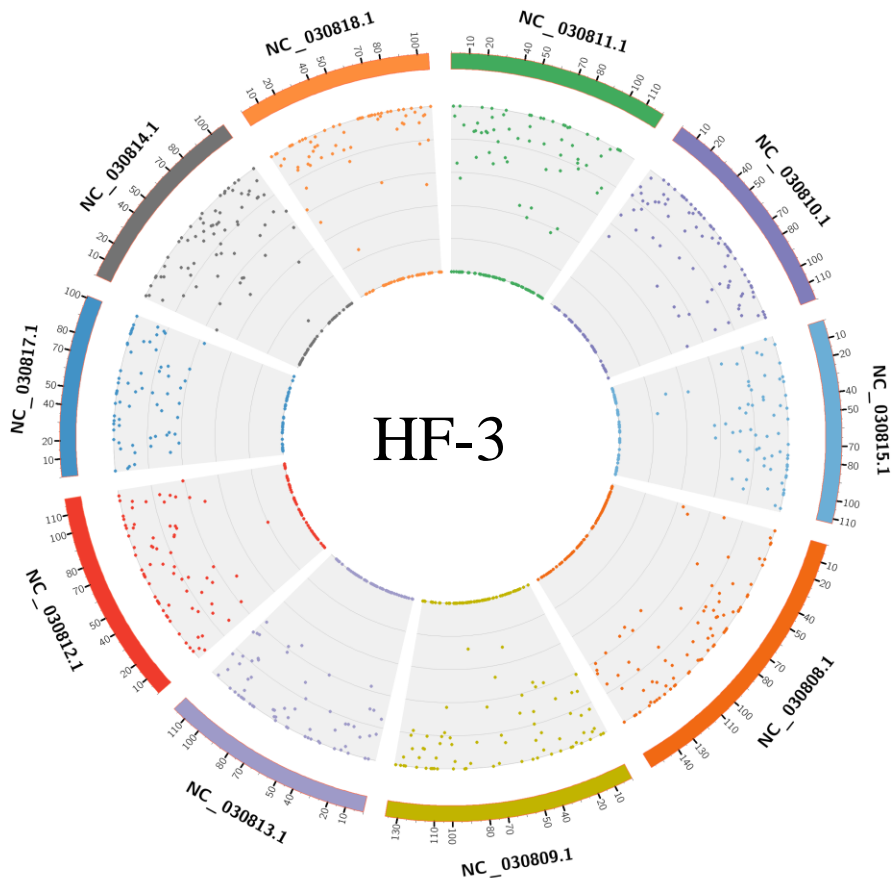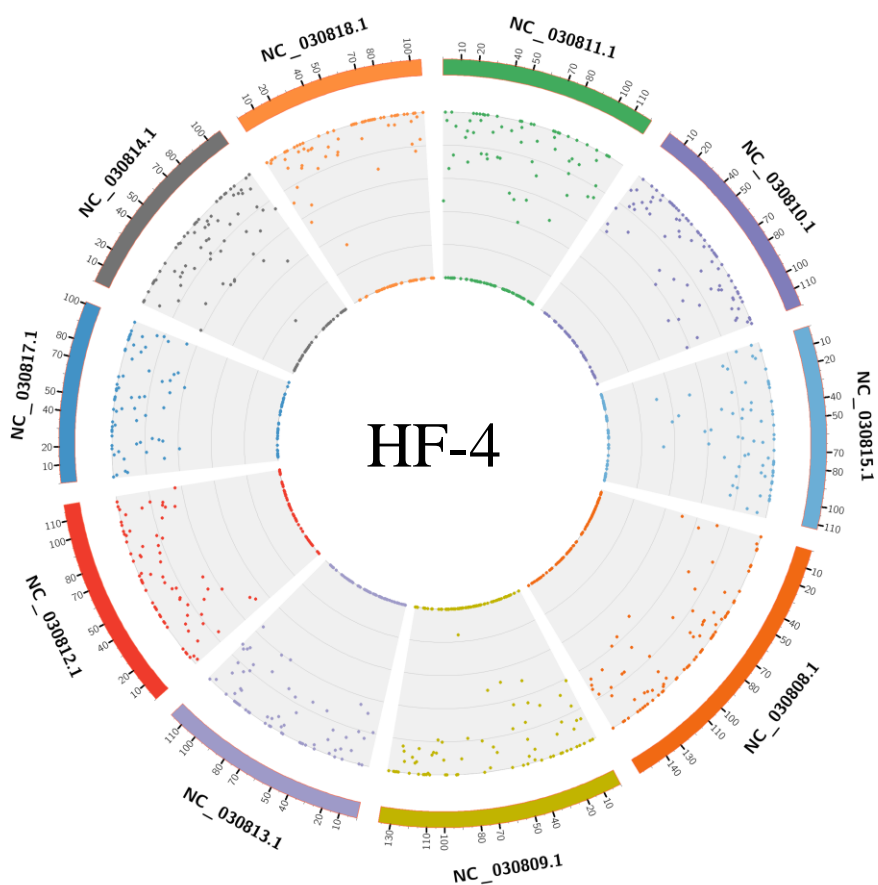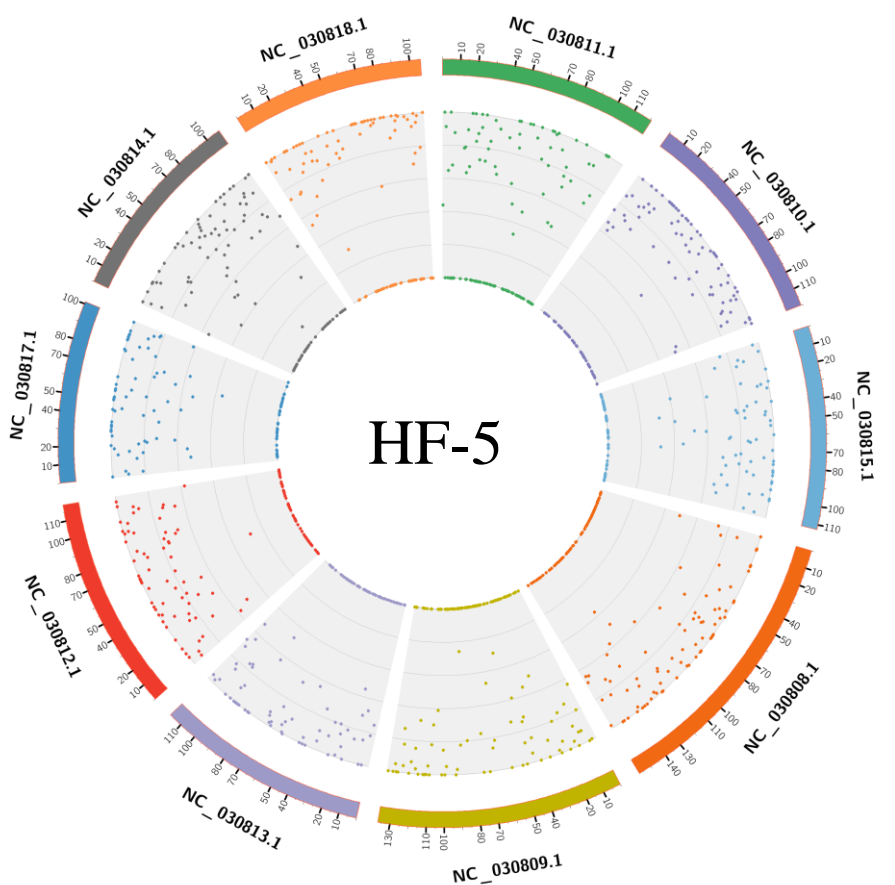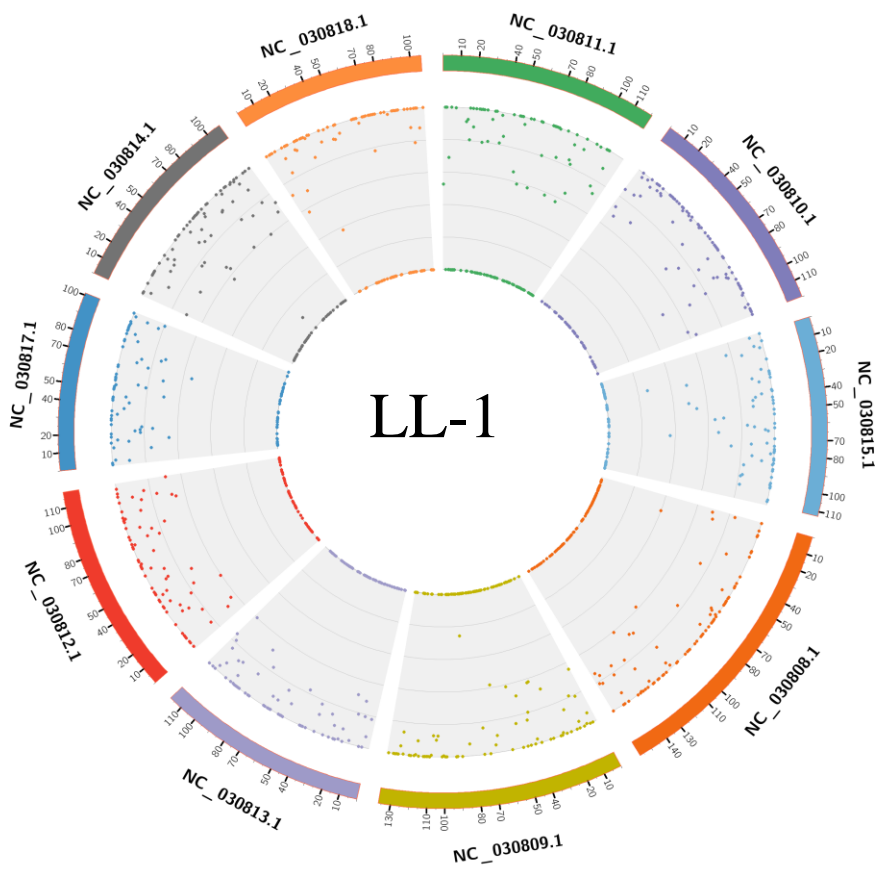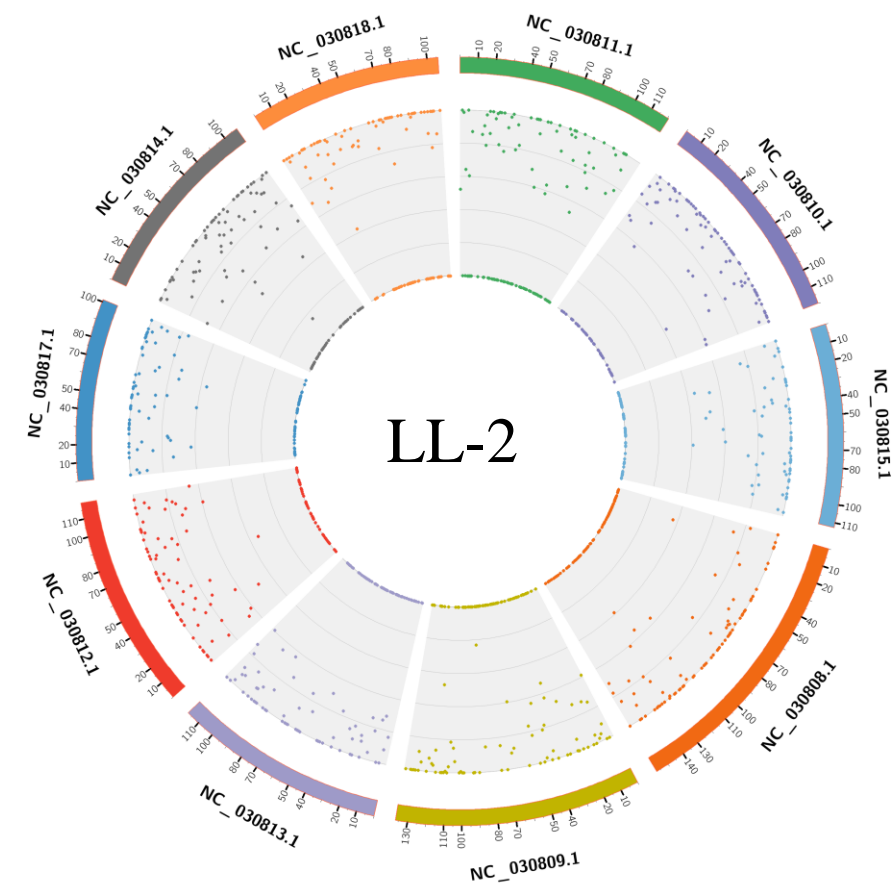

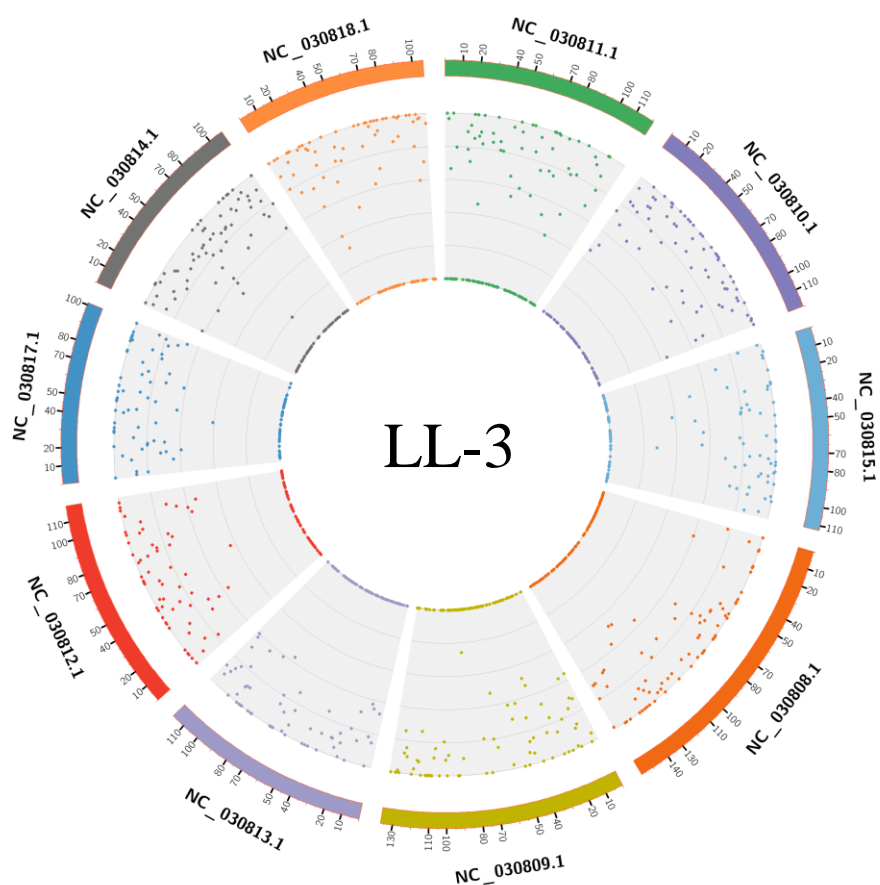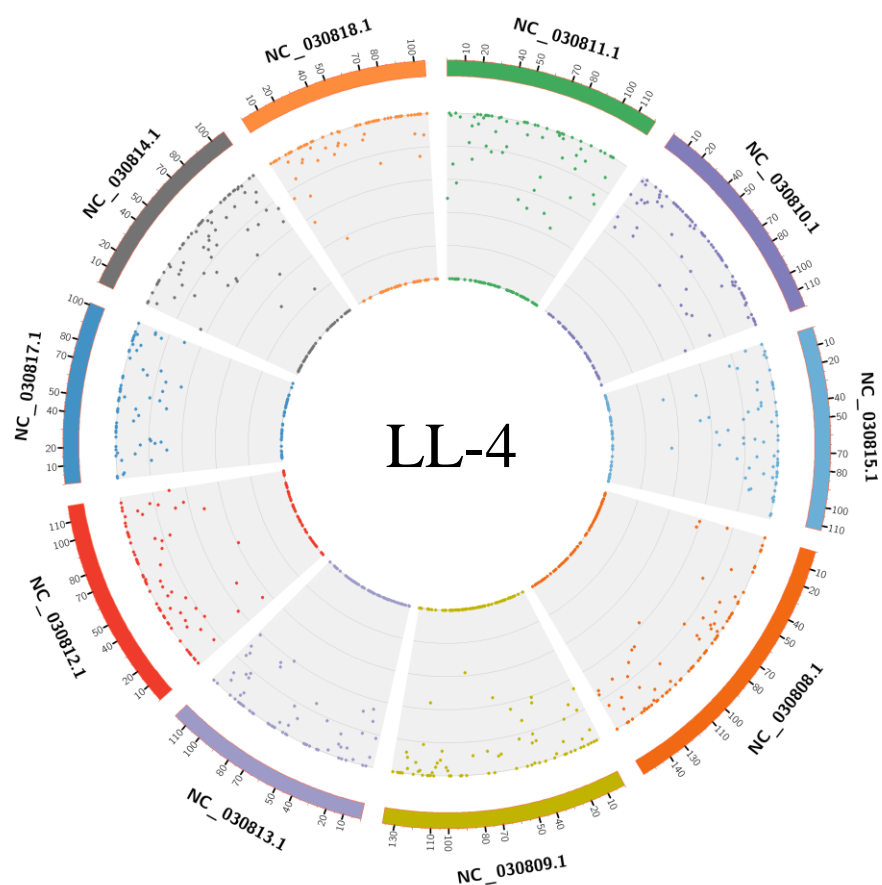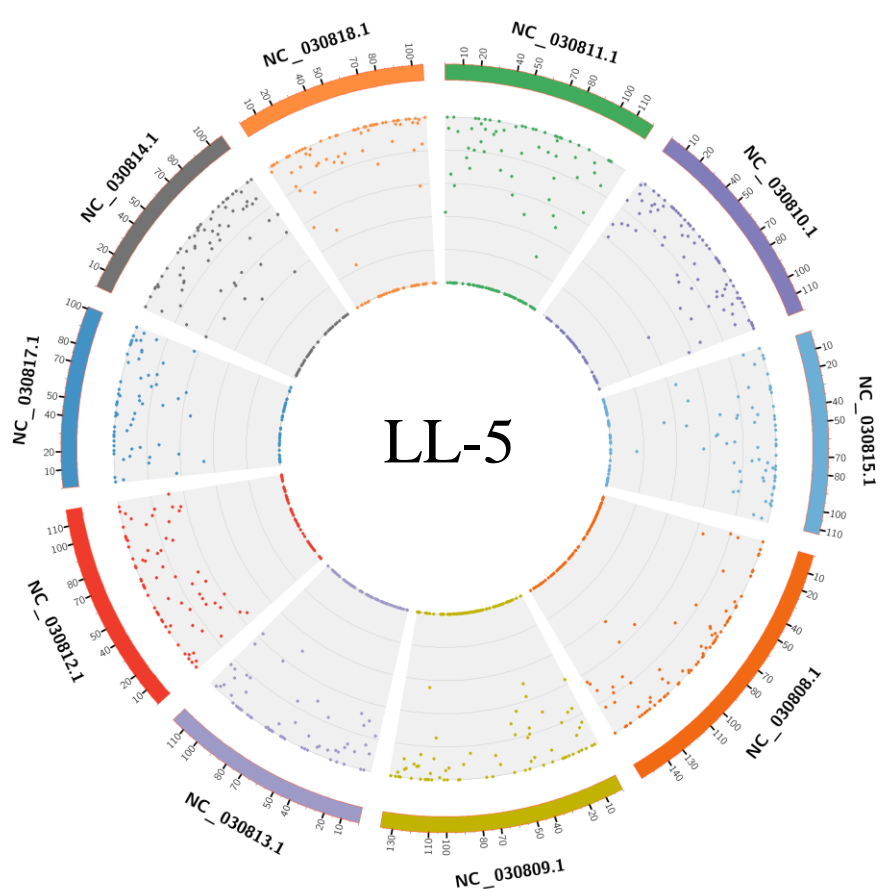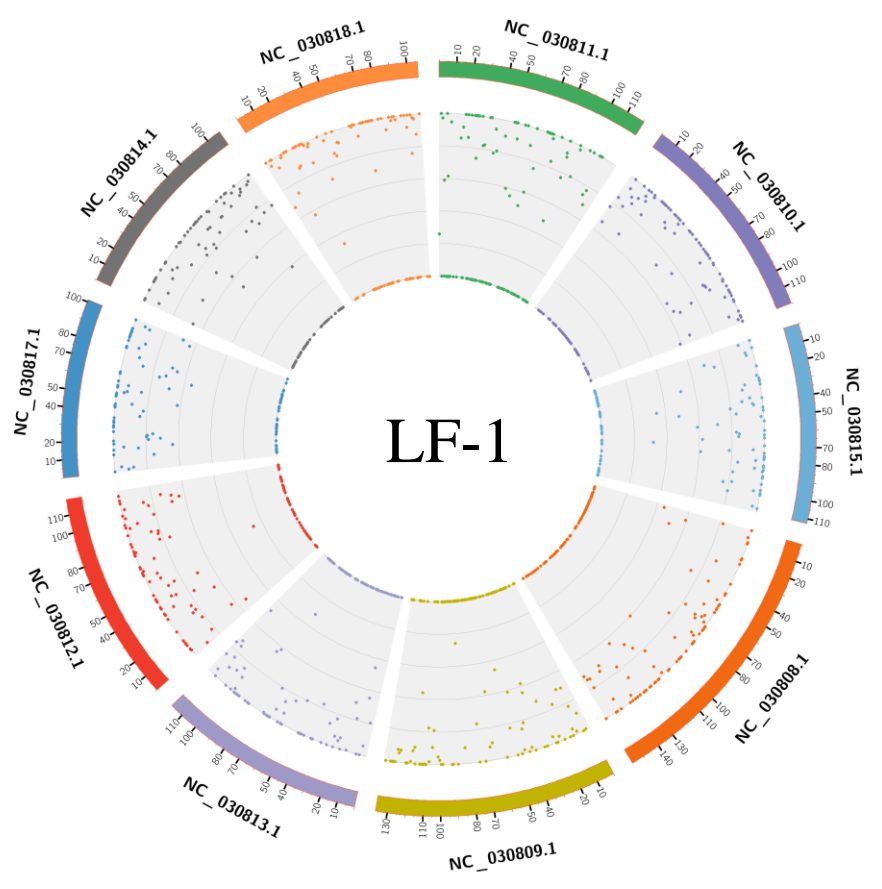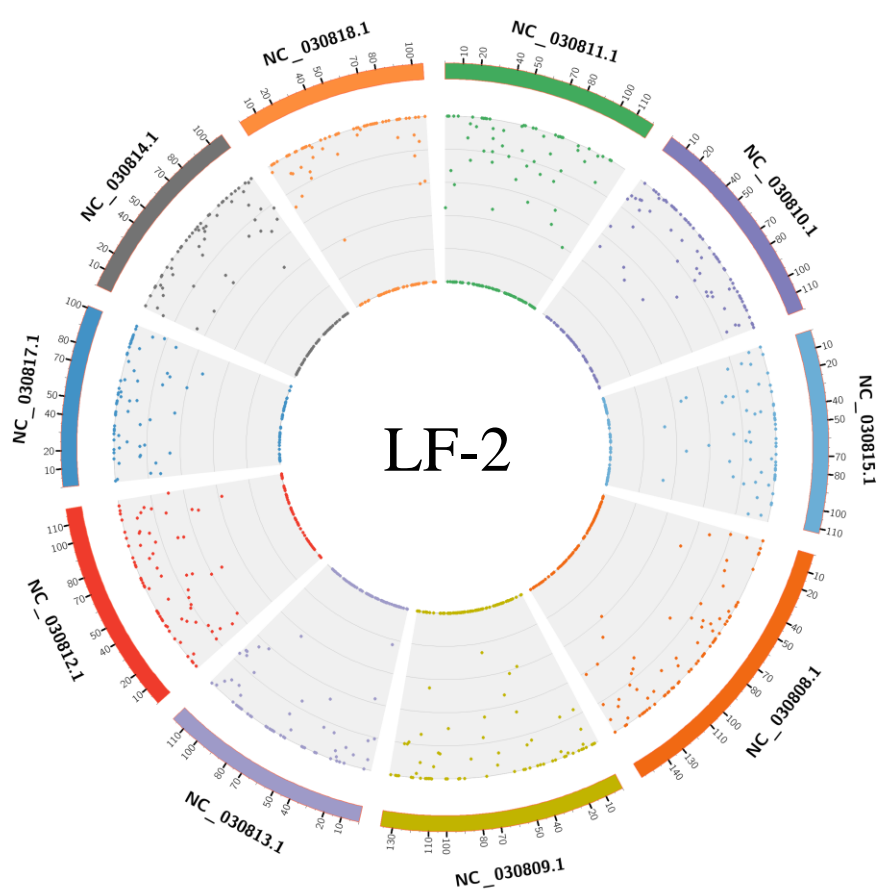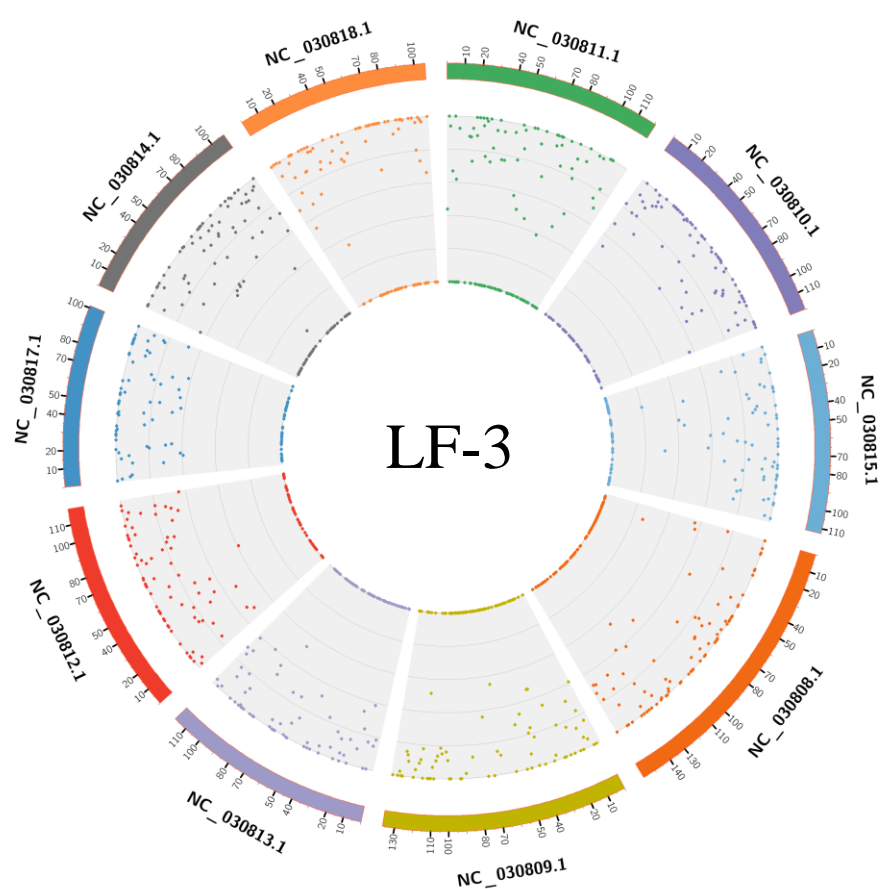

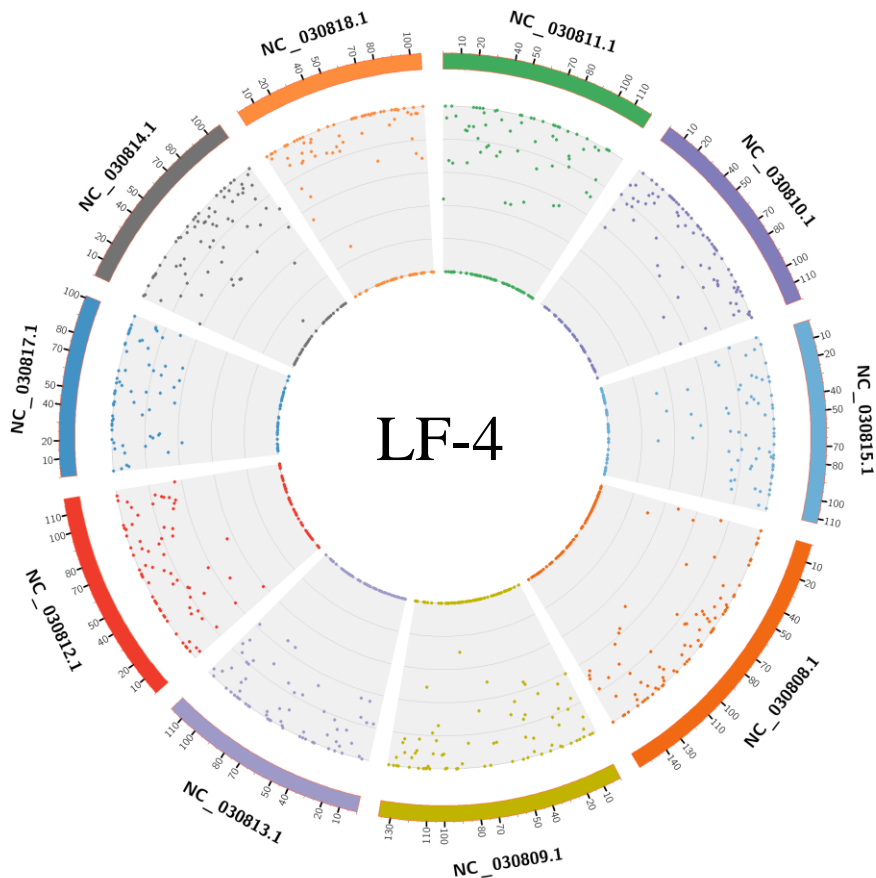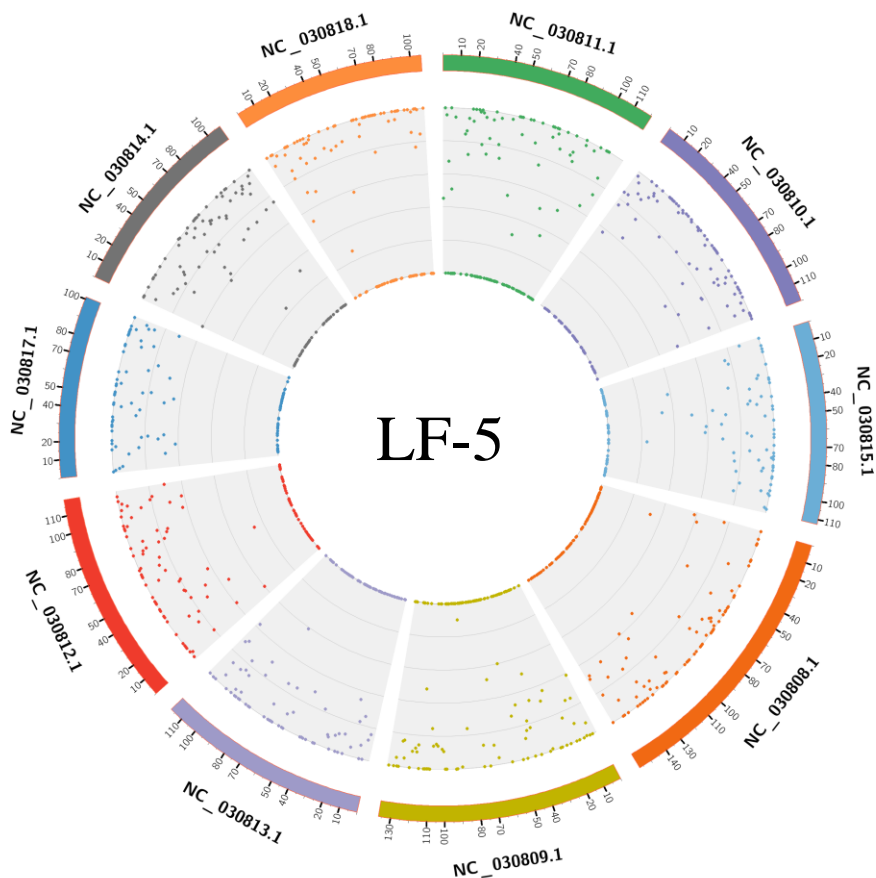

Supplement: Supplementary file 2 [file DataSheet1.PDF]
